# Acquired methemoglobinemia induced by dapsone in a 16-year-old female: A case report from Iraq—A case report: Retraction

**DOI:** 10.1097/MD.0000000000044335

**Published:** 2025-08-22

**Authors:** Rusul Mahdi Abid, Muthanna Thiab Fendi, Hashim Talib Hashim, Zainab Ali Hussein, Ashraf Fhed Mohammed Basalilah

The article “Acquired methemoglobinemia induced by dapsone in a 16-year-old female: A case report from Iraq—A case report”^1^, which appears in Volume 104, Issue 30 of *Medicine*, is being retracted. Information brought to our attention during a recent investigation casts doubt on both the authorship and credibility of the presented case, and the Publisher has lost faith in the integrity of the content.
